# Money matters: a critique of ‘informed financial consent’

**DOI:** 10.1093/medlaw/fwae015

**Published:** 2024-05-09

**Authors:** Sara A Attinger, Ian Kerridge, Cameron Stewart, Isabel Karpin, Siun Gallagher, Robert J Norman, Wendy Lipworth

**Affiliations:** Faculty of Medicine and Health, Sydney School of Public Health, Sydney Health Ethics, The University of Sydney, Sydney, Australia; Department of Philosophy, Macquarie University, Macquarie Park, Australia; Faculty of Medicine and Health, Sydney School of Public Health, Sydney Health Ethics, The University of Sydney, Sydney, Australia; Department of Philosophy, Macquarie University, Macquarie Park, Australia; Haematology Department, Royal North Shore Hospital, St Leonards, Australia; Sydney Health Law, Sydney Law School, University of Sydney, Sydney, Australia; Faculty of Law, University of Technology, Sydney, Australia; Faculty of Medicine and Health, Sydney School of Public Health, Sydney Health Ethics, The University of Sydney, Sydney, Australia; Robinson Research Institute, Adelaide University, Adelaide, Australia; Faculty of Medicine and Health, Sydney School of Public Health, Sydney Health Ethics, The University of Sydney, Sydney, Australia; Department of Philosophy, Macquarie University, Macquarie Park, Australia

**Keywords:** Consumers, Costs conversations, Disclosure, Financial transparency, Informed consent, Informed financial consent

## Abstract

In recent years, concerns about the financial burdens of health care and growing recognition of the relevance of cost to decision making and patient experience have increasingly focused attention on financial ‘transparency’ and disclosure of costs to patients. In some jurisdictions, there have been calls not only for timely disclosure of costs information, but also for ‘informed financial consent’. However, simply putting the ‘financial’ into ‘informed consent’ and invoking an informed consent standard for cost information encounters several ethical, legal, and practical difficulties. This article will examine the viability and desirability of ‘informed financial consent’, and whether it is possible to derive ideas from traditional informed consent that may improve decision making and the patient experience. We argue that, while there are important legal, ethical, and practical challenges to consider, some of the principles of informed consent to treatment can usefully guide financial communication. We also argue that, while medical practitioners (and their delegates) have an important role to play in bridging the gap between disclosure and enabling informed (financial) decision making, this must be part of a multi-faceted approach to financial communication that acknowledges the influence of non-clinical providers and other structural forces on discharging such obligations.

## I. INTRODUCTION

Patients need information in order to make informed decisions about their health care. While it has long been recognised that this needs to include information about physical and psychological risks and benefits, policymakers, physicians, health economists, and bioethicists have become increasingly concerned about the financial burden of healthcare on patients.^1^ Even in contexts with substantial publicly funded healthcare systems, such as Australia, patients may receive government subsidy for some, but not all consultations, treatments, and tests (which may be partially subsidised or not subsidised at all, and may or may not be covered by private insurance). This means that patients may have to face financial uncertainty, surprises, and difficult financial choices. Concern about financial burden has translated into a perceived need to include information about pricing and costs of healthcare in processes of communication and shared decision making.^2^

There is variation in how different jurisdictions have responded to this need. In the USA, for example, this has primarily taken the form of calls for greater financial ‘transparency’,^3^ with an emphasis on pre-commencement disclosure of prices or costs. There have also been references to costs and financial information in the contexts relating to informed consent to medical treatment, with some scholars and policymakers looking to the doctor–patient relationship as the historical ‘locus of disclosure’.^4^ In Australia, the Medical Board of Australia’s *Good Medical Practice Code of Conduct* stipulates that patients should be informed about fees and charges ‘in a timely manner to enable them to make an informed decision about whether they want to proceed’^5^ and that doctors should advise patients where there may be additional costs when referring for investigation, treatment, or a procedure.^6^ In the UK, General Medical Council guidance provides that doctors should give information about any out-of-pocket costs as part of informed consent to treatment.^7^ In New Zealand, the *Code of Health and Disability Services Consumers’ Rights* creates a right for costs associated with healthcare to be ‘fully explained’.^8^ Of course, approaches to costs communication are likely to be influenced by differing systems of healthcare funding and magnitudes of out-of-pocket costs to patients in each jurisdiction. In some settings, there have been calls not just for disclosure of information, but for so-called ‘informed financial consent’. This has received academic attention,^9^ and has been advocated by professional bodies^10^ and policymakers^11^ in some jurisdictions.

In Australia, as part of government initiatives in the early 2000s,^12^ ‘informed financial consent’ emerged as a political response to rising out-of-pocket costs for individuals who have private health insurance but still need to pay sometimes substantial ‘gap’ costs above their coverage. In hospital settings, statutory requirements mandate disclosure of out-of-pocket costs before undergoing treatment, and describe this as ‘informed financial consent’.^13^ Use of ‘informed financial consent’ has also been advocated and affirmed by the Australian Medical Association (AMA) in concert with the Australian Government Department of Health in the form of voluntary guidance for medical professionals released in 2019.^14^ While the AMA statement does not support a legal obligation to obtain informed financial consent,^15^ medical professional disciplinary proceedings have invoked ‘informed financial consent,’ for example, to indicate the necessity for disclosure of lower cost alternatives in the public system for private patients.^16^ There has also been a limited development of the concept in contract law regarding provision of care involving public subsidy.^17^ In interpreting a contested contract for services between an anaesthetist and a patient, one Australian court decision addressed the absence of provider disclosure of private fees as absence of agreement to a price term.^18^ The court implied a reasonable fee at the standard rate of public subsidy for the service (rather than the higher private fee).^19^ This decision has also been referred to as establishing a legal basis (albeit limited) for ‘informed financial consent’ in contract law.^20^

The idea of ‘informed financial consent’ has also received some academic attention.^21^ These discussions posit to varying degrees that financial information-giving should be part of informed consent to treatment. In the USA, scholars have proposed ‘informed financial consent’ primarily in the form of advance costs disclosure in an environment of minimal transparency,^22^ viewing the introduction of the *No Surprises Act* by US Congress in 2020 as a limited but ‘meaningful nudge’ towards informed financial consent.^23^

Invocations of ‘informed financial consent’ have, however, come without clear or stable definitions of the concept or express recognition of its limitations—even within jurisdictions such as Australia where the concept has been explicitly (albeit unevenly) adopted into health policy and regulation. For example, some policymaking has equated ‘informed financial consent’ with mere receipt of information,^24^ while others have alluded to obligations to provide something more than costs disclosure.^25^ Guidance from the AMA about informed financial consent primarily focuses on pre-commencement episode-based disclosure, although it does recommend discussing a patient’s ability to pay for surgical or other medical interventions (including ongoing consultations),^26^ shifting focus somewhat towards a patient’s likely, holistic pathway. This can be contrasted to informed consent to treatment, where there is an emphasis on patient understanding and dialogue between doctor and patient, including about alternative options and contingencies, and ongoing discussions as investigations and treatments progress and evolve.^27^ Calls for doctors to provide information about costs also do not substantially consider the practical barriers that patients and practitioners face accessing and communicating about financial information.^28^

In this article, we argue that there are important ethical, legal, and practical considerations that limit the viability and desirability of ‘informed financial consent’ as a complete approach to financial communication in the medical context. There are, however, some elements of informed consent that can usefully be applied to financial communication in order to bridge the gap between mere disclosure and promoting informed decision making. This has particular resonance when care is not an emergency (so conversations can be had) and where care is open-ended and complex.

In Section II, we describe the key justifications for financial transparency in health care. In Section III, we discuss the arguments for, practicalities of, and challenges of applying ‘informed financial consent’ to communication about the financial aspects of care. We suggest that while the concept has important limitations, it is possible to derive ideas from informed consent that could improve decision making and the patient experience. In Sections IV and V, we conclude that practitioners (and their delegates) have an important role to play in bridging the gap between disclosure and enabling informed (financial) decision making, but that this must be part of a multi-faceted approach that acknowledges the influence of non-clinical providers and other structural forces on discharging such obligations. We consider some key elements of a multi-faceted, consent-oriented approach to financial communication in health care.

While healthcare systems will have different demands upon and barriers to good costs communication, we focus here on the Australian context that has developed notions of ‘informed financial consent’ in ethics, law and policy.^29^ The Australian approach is also internationally influential and has been referred to approvingly by scholars in the USA.^30^

## II. JUSTIFICATIONS FOR FINANCIAL TRANSPARENCY

Demands for financial transparency in health care have been driven primarily by the increasing recognition of the financial burden of health care on patients and the knowledge that patients do sometimes, in fact, base their treatment decisions on cost.^31^ Some of the effects of these costs include patient non-adherence to treatment^32^ and ‘financial toxicity’ (harms to patients resulting from financial stressors).^33^ Importantly, these problems arise even in countries with substantial public healthcare and insurance systems, such the UK and Australia, where care is free at the point of delivery for certain treatments. Even in these jurisdictions, there may be out-of-pocket costs, because some services are not fully covered, and some are excluded from public subsidy, and patients receiving entirely ‘free’ care to begin with may require additional interventions or referral in future with associated costs. In an attempt to mitigate some of the effects of unexpected costs, scholars, policymakers, practitioners, and consumer advocates have highlighted the need to ensure that (outside of emergency situations) patients receive timely information about costs to prevent ‘bill shock’ and enable informed decision making about care.^34^

The need for financial transparency is also justified on a number of moral grounds. Most fundamentally, it is seen as a way of respecting patients’ autonomy, avoiding exploitation, fulfilling the obligation of veracity, and actualising the goals of shared decision making.^35^ Financial transparency is also seen by some to respect patients’ choices as ‘consumers’ of healthcare goods and services.^36^ The notion that patients are consumers (rather than passive recipients of care) places more responsibility and accountability on patients to seek information, and correspondingly, places a strong obligation on healthcare organisations and physicians to be transparent.^37^ In placing responsibility on patients, a side effect of transparency is reducing professional and provider liability.

Financial transparency is seen as a means of a managing providers’ financial conflicts of interest (by making financial interests visible to patients), managing unequal access to information between producers and consumers (ie, ‘information asymmetry’) and ensuring the effective functioning of healthcare markets.^38^ The latter requires patients as ‘consumers’ to be able to access relevant information upon which to make their decisions. Indeed, requiring pre-treatment disclosure of costs brings the purchase of medical services into line with most other economic transactions.

Financial transparency has also been advanced as means of ensuring provider accountability^39^ and resource stewardship^40^ and countering price inflation in medical marketplaces by creating competitive market price pressure.^41^ In this regard, it is significant that both patients and health professionals have rapidly increasing access to online tools^42^ that seek to facilitate both the provision and comparison of financial information.

Finally, it is argued that financial transparency can address, to varying degrees, several legal obligations and norms that govern health and medical care, relating to contracting, consumer protection and fair trading, consent and risk disclosure, health practitioner professional responsibility, and fiduciary obligations. For example, transparency may contribute to the conditions for forming a valid contract and provider disclosure is incentivised where a court’s approach to contract construction would imply a lower price.^43^ Transparency also fulfils some of the requirements of consumer protection regulation, which seeks to incentivise or mandate disclosures that producers and providers might be reluctant to divulge.^44^

## III. INFORMED FINANCIAL CONSENT

There are several ways in which financial transparency can be achieved and, as noted above, several ways it has been interpreted and instantiated around the world. The most basic and least demanding form of financial transparency is *price disclosure*, which provides information about the standard price of a health good or service. This may take the form of list prices or a ‘menu’ of services. While price disclosure is relatively straightforward, it has been argued that this is not sufficient in healthcare and that information needs to be specific to the individual patient.^45^*Cost disclosure* is therefore preferable because it provides information about the amount that a patient will likely pay out-of-pocket for a service. This may take the form of a medical bill that includes out-of-pocket cost to an individual patient after taking into account applicable insurance coverage. Another approach towards financial transparency goes further than cost disclosure, and applies the notion of ‘informed consent’ to financial communication about the financial aspects of care. As noted earlier, however, calls for ‘informed financial consent’ are often lacking in clear justifications for invoking the concept of informed consent, explanations for how to enact it, and recognition of its limitations. Here, we address these lacunae by: (i) summarising the general functions that consent has in ethics and law, which could also be applied to financial communication; (ii) considering what genuine ‘informed financial consent’ would entail; and (iii) considering whether it is achievable given the legal, ethical, and practical challenges such an approach raises.

### A. *Functions of informed consent*

The potential justifications for informed financial consent are similar to the justifications for consent more generally in ethics, law and professionalism, where the concept serves three main functions.

The first is a *permissive* function, where consent processes formalise the way that permission is granted to touch and/or treat a patient’s body or mind. This normally requires the patient to understand basic information about the nature of the health intervention.^46^

The second function is a *risk* function whereby consent processes are used to provide information about material risks to patients to help them make decisions about treatment.^47^ The level of information that needs to be provided about risk may vary depending on the patient’s desire for information and their personal assessments about what risks are material to them.

Both the permissive function and the risk function are concerned with ethical values of autonomy and respect for persons and those ethical values are also featured in legal accounts of consent, such as via the tort of battery, the crime of assault and claims for negligent advice in medical treatment (*informed consent in negligence*).^48^ The practical effects of compliance with these legal standards is a reduction in liability and a measure of control by the patient over the risk they wish to undertake.

A third function of consent is a *relational* function, where the consent process provides a framework for the therapeutic relationship, in which relationships of trust and understanding may develop within the power dynamics of patients, families and health professionals.^49^ The relational function of consent is featured in ethical discussion of relational autonomy and in ethical discussions about hope, trust and power.^50^ Legally, the relational function is featured in discussions around fiduciary duty, unconscionable transactions, undue influence and the question of adequate disclosure of conflicts of interest.

All of these functions, alongside the justifications for financial transparency discussed earlier, could potentially be used to justify the provision of financial information as part of consent (either viewing this as part of consent to treatment or as an additional consent requirement).

### B. *Requirements for valid informed financial consent*

As discussed above, the risk function of consent represents an individual’s autonomous authorisation of a medical intervention, based on dialogue between doctor and patient about a proposed treatment, alternative options, including non-treatment, and the risks and benefits of each.^51^ A true informed financial consent standard would, therefore, require doctors to consider both what up-front costs and initial treatment costs are likely to be, and what additional financial information would be required. For example, if the patient is likely to have a complex clinical and therefore financial trajectory, or if their clinical outcomes are likely to be impacted by the financial decisions that they make, such as by delaying their treatment. It would also require that information is given not only about the financial implications of a recommended treatment but also about the costs of alternatives (including the costs of not proceeding with treatment at all). Discussions of cost would also need to be integrated with discussions about clinical risks and benefits^52^ so that patients can determine not only what they can afford, but also what represents value for money to them. In order for informed financial consent to be valid, at least from an ethical perspective, information would have to address patients’ unique circumstances, conversation would elicit patients’ values and preferences (including regarding finances), and some attempt would need to be made to ensure that patients sufficiently understand the information provided and have had the opportunity to ask questions.^53^

### C*. Challenges of applying informed consent to financial communication*

Despite efforts to invoke and operationalise the concept of ‘informed financial consent’,^54^ there are several challenges to doing so, which may explain why it has not been uniformly supported or translated into policy and practice. Some of these—such as the difficulties with knowing or predicting costs—apply to any kind of financial transparency, while others are specific to an informed consent standard.

#### 1. *Challenges that apply to any kind of financial transparency*

One of the main challenges for any approach of financial communication—whether transparency through pre-commencement disclosure or as part of an informed consent framework—is the difficulty of knowing the full costs of an individual’s treatment journey at the time of the key decision points. While providing information about standard prices (price disclosure) is relatively straightforward, it has been argued that this is not sufficient and that information needs to be specific to the individual patient.^55^ Providing patients with information about personal costs can, however, be difficult to achieve in practice.^56^ This is because healthcare costs are often complex, unbundled (based on a procedure rather than an episode of care) or generically bundled (where a single price covers a group of separate procedures commonly performed together), and unpredictable at the start of a course of treatment.^57^ Thus, it can be difficult for pre-commencement costs disclosure to be accurate and comprehensive. Meaningful cost disclosure is particularly difficult for treatment that is open-ended and occurs in cycles over extended periods of time (such as cancer treatment, assisted reproductive therapy, or management of chronic disease).

Even for so-called ‘shoppable’ services (non-urgent care that is time-limited and can be scheduled in advance), price or pre-commencement costs disclosure will not always be sufficient to deliver meaningful personal costs transparency.^58^ For example, a person considering in vitro fertilisation (IVF) may be able to compare a generic per-cycle price of IVF among providers, but price listings do not tell them how many cycles of treatment a patient like them will likely undertake, how much of certain drugs they may require, and what aspects of treatment might be ‘must have’ versus ‘nice to have’. Further, the basic per-cycle price of IVF may not be standard in what it includes and excludes, making meaningful comparison difficult.^59^

Additional barriers to both price and cost transparency include the difficulty of generating lists of standard prices when a wide variety of services are offered—particularly where these services involve third-party payers or providers, individual insurance coverage, and a variety of specialists working together, each with different billing structures. Some pricing structures may also mean that personalised cost information might only be available after treatment consultation, when the patient has already ‘invested’ in a provider (ie, it can cost money to get information about treatment costs). Doctors may also have financial interests in the products and services that they provide,^60^ which may influence their ability and willingness to discuss issues of cost.

These challenges are not unique to the healthcare setting, and there are numerous common scenarios, such as house renovation or building, where it may be difficult to predict precise costs. Standard approaches to imprecise costs disclosure in contracts (for eg, trade services) do not, however, necessarily provide a good model for communication in medical care. Further, in hybrid or mixed public and private payer health systems, patients may not be accustomed to paying for complex treatment (and thus complex billing) or, indeed, for any treatment at all, or may not anticipate the financial considerations of commercial providers that can influence treatment offering, such as the profitability of some treatment options compared to others, or to non-treatment. Additionally, in Australia, there is commonly a publicly funded component to private care, which distinguishes contracts for medical services from purely private transactions.

#### 2. *Challenges of communicating about finances through ‘informed financial consent’*

In addition to there being challenges that apply to any kind of financial transparency, there are also additional challenges to applying informed consent as an approach to financial communication. For, as much as financial communication through ‘informed financial consent’ seeks to adopt the strengths of an informed consent framework, it also assumes some of the difficulties of informed consent based on information about (non-financial) risks and benefits, exacerbates these difficulties and creates new ones.

Some of the ongoing challenges of any kind of informed consent to treatment include knowing how to define materiality and minimum standards of information provision, and ensuring that decision making is genuinely ‘shared’ when communication is not always easy and time is often limited.^61^ There is ongoing legal uncertainty about informed consent to treatment. For example, materiality is often determined subjectively to some degree, and that will tend to turn on the circumstances of a particular case. In this regard, it is important to bear in mind that even if costs can be predicted and clinicians are willing to discuss them, individuals’ financial situations may change over time, so the financial implications of ongoing treatment, and the relevance of finance as a decision-making factor, cannot be presumed from disclosure of likely costs before commencement. It can also be difficult to determine what a ‘reasonable patient’ would consider to be material, though there are arguably some common components of materiality. These ethical and legal challenges in informed consent to treatment would likely be replicated, or even exacerbated if demands were placed on clinicians to obtain genuine informed financial consent. For example, can a doctor know what is significant to someone in terms of their finances? Determining this is difficult enough when it comes to physical and psychological risks and benefits, but these are at least concerns that fit within the general area of expertise of healthcare professionals. The same is not true of financial risks, harms and benefits, which could require an understanding not only of a patient’s values but also of their insurance coverage, their other (competing) financial commitments and their various financing options (eg, accessing retirement funds, asking family or friends, crowdsourcing, etc).

The requirement for informed financial consent is also complicated legally by questions about whether medical practitioners are under a fiduciary duty to their patients (ie, whether they have to act in the best interests of their patients). In the USA and Canada where doctors are presumed fiduciaries, this implies an obligation not just to disclose costs but also to inform them about potentially competing or conflicting financial interests.^62^ In other jurisdictions such as England, Wales, Australia, and New Zealand, medical practitioners are not presumed fiduciaries and patients need to be able to prove that their doctors owed them a duty to disclose financial information and conflicts of interest. Irrespective of whether doctors are fiduciaries or not, what doctors discuss, and how they discuss it, may vary according to their own financial interests.

There is also a risk that if doctors attempt to engage more closely in the financial aspects of an individual’s care, this may engender incorrect assumptions about what people can afford which, in turn, may limit what treatment options are discussed with them. In this regard, it is noteworthy that the financial status of patients (assumed or otherwise) has been shown to influence health professionals’ decision making even in contexts where patients are not paying directly for their care (ie publicly or privately insured).^63^ Similarly, in user-pays contexts, concerns have been raised about the balance between not providing a service, which may be discriminatory or prejudicial, versus the potential harm of offering treatment to a patient beyond their financial means,^64^ particularly where treatment has low chances of success. On the other hand, for patients to share in decision making and be fully informed about the risks and benefits of their options, informed consent to treatment may need to encompass discussion with a patient about their financial constraints, especially where options offer similar clinical results. While a patient’s expressed concerns about finances may go to materiality of information under informed consent, some objective indicators such as insurance status will also be relevant because of the predictable impact on patient out-of-pocket costs.^65^

Another challenge with informed financial consent is that it requires that information be given not only about the financial implications of recommended treatment but also about the costs of alternatives (including the costs of not proceeding with treatment at all). In mixed health systems, consent in the private setting would need to include advising on the availability of the treatment in the public system.^66^ But while it is reasonable to expect healthcare professionals to know about the physical and psychological risks and benefits of alternative treatments (or of no treatment), it is arguably not always reasonable to expect healthcare professionals to have a complete knowledge of the costs associated with alternative clinical decisions or with care offered by other practitioners. For example, a private billing orthopaedic surgeon may not be expected to know the detailed costs charged by other privately billing surgeons for the same knee replacement procedure. Competition law might also prohibit discussing prices with competitors to prevent price-fixing. Arguably, however, it would be reasonable for a practitioner to be able to discuss, at least in ball park figures, the relative expense of alternative pathways that are clinically appropriate, particularly where there is high potential financial impact in undergoing treatment.^67^

Additionally, it is difficult, if not impossible, to know where to draw the line about what counts as a cost. For some interventions, ‘direct’ costs may be definable and able to be costed. For example, the Medical Board of Australia provides guidelines for the range of costs required for informed (financial) consent in cosmetic surgery, including costs of other practitioners involved in care and possible costs of further, consequential treatment.^68^ Importantly, these guidelines stipulate that if costs of third party treatment providers are not known, a practitioner should provide indicative costs and at the least, direct the patient on how to obtain that information.^69^ Yet, decisions about care may entail a broader calculus. For example, a clinician might not be reasonably expected to calculate indirect costs such as the costs of travelling for attending medical care or the costs of taking time away from work or needing to pay for childcare. Thus, while some advocate for costs disclosure to be inclusive of a broader range of costs,^70^ it is not clear what can be reasonably expected of clinicians in providing this information because of the degree to which indirect costs are opaque, unknown, complex, and variable according to patients’ circumstances. That said, there may be some clinicians who can be expected to know and provide information about indirect costs. For example, a clinician who routinely works with patients in rural or remote areas may be expected to have and convey information about the comparative expense between local provision and travelling to a metropolitan area.

In terms of the elements that make informed consent valid, for informed financial consent to meet the standards of informed consent more generally, clinicians would need to ensure that patients have the capacity to make decisions about their own health care, and understand information disclosed to them about their healthcare costs. This may, however, be an unrealistic expectation for financial information. While it seems reasonable for healthcare professionals to have skills in assessing patients’ capacity and understanding of information about physical and psychological benefits, it may be unreasonable to expect them to assess patients’ financial literacy.

More generally, informed financial consent poses the risk of blurring the boundary between informing patients about costs and providing financial advice. In addition to providing information about costs, clinicians may go further, discussing the availability of financing options, such as personal loans, buy-now-pay-later schemes, early access to retirement funds^71^ or financial aid programs. Not only is providing advice about certain financial products subject to restrictive regulation (such as licensing requirements in Australia^72^), but it is also likely to create ethical conflicts for practitioners. In cosmetic surgery in Australia, where patients typically bear high out-of-pocket costs, ethical guidelines preclude practitioners from recommending or offering commercial financing schemes, but they may inform patients about such schemes as accepted payment methods.^73^ While the question ‘which clinical option is most cost-effective for my desired outcome?’ will not ordinarily constitute financial advice, it may involve assisting with a broader calculus which may be difficult or inappropriate for clinicians. These difficulties may point to the need for independent financial expertise to help patients navigate personal finance.

From a more pragmatic perspective, informed financial consent requires that both patients and clinicians are willing to engage in this discussion, which might be difficult for cultural or contextual reasons, or because one or both parties feel uncomfortable doing so.^74^ Patients may, for example, view finance as a private matter and/or a personal responsibility, or prefer to spend consultation time discussing the clinical aspects of treatment. Patients may fear being denied treatment or not being offered all available options should they express concerns about the costs of care or might be concerned about receiving lower quality care as a result.^75^ Doctors, in turn, may be uncomfortable or unwilling to discuss costs with their patients because of general societal norms that dictate that this is a personal issue and/or not part of a clinical interaction between a patient and a doctor. Doctors may also be uncertain about how they should respond to financial information that is shared with them or the boundary with giving financial advice, as discussed above. Doctors may not have familiarised themselves with financial information or may not be trained in managing costs communication. Particularly in complex treatment contexts, costs discussions may also be time consuming. When consultation time is limited, doctors may prefer to focus on or prioritise providing their clinical expertise. While some communication support tools have been developed in an effort to overcome some of these challenges, they are not a panacea. We discuss these tools, alongside other strategies in Section IV.

There are additional legal explanations for why informed financial consent has not been uniformly supported or translated into policy and practice. In many jurisdictions, legal duties are grounded in the law of negligence and governed by concepts of materiality, meaning that valid informed consent requires communication of information which is likely to influence a patient’s treatment decision. But legal duties focused on disclosure of material risks tend not to explicitly include information other than that pertaining to the physical and psychological risks of interventions.^76^ For example, in Australia medical practitioners have a duty in negligence to warn patients of material risks associated with a proposed treatment, but financial risk is not one of the classes of risks that have so far been recognised at common law.^77^

It is, however, conceivable that financial risks will come to be considered a type of material risk as there has been shift over time from profession-based determinations of materiality (professional practice/the ‘reasonable professional’) to more patient-centric approaches, which focus on what a ‘reasonable patient’ or what a ‘reasonable person’ in position of the particular patient would like to know, including risks, side effects, and alternatives.^78^ The materiality of costs information might also come to be captured in the *objective* aspect of materiality, rather than needing to be raised as a significant factor by a patient in order to trigger a subjective standard of materiality. This idea that costs are an objective, or presumed, aspect of materiality is consistent with the trend towards patients being viewed as ‘consumers’. It has also been argued that ‘financial toxicity’ should be framed as clinical risk or ‘side effect’ of treatment,^79^ and so could be just as material as any other effect of treatment. Together, these shifts in thinking about materiality and decision making could shift the balance legally, such that financial information could come to be considered (objectively or subjectively) material. In Australia, a jurisdiction with a legal basis for informed consent under negligence, the materiality of financial information has not been legally tested.

## IV. CONVERSATIONS ABOUT MONEY

Given all these challenges, it may be a mistake to attempt to address the need for financial transparency and the limitations of mere disclosure by simply demanding ‘informed financial consent.’ At the same time, it seems mistaken to think that clinicians have *no* obligations beyond mere disclosure regarding costs information and to ignore what *can* be translated from informed consent to treatment. What is needed is an approach to communication that draws more consistently and substantially on consent to treatment—regardless of whether it is called ‘consent’ or not, or is considered part of consent to treatment or a separate obligation. The question of informed financial consent suggests an important patient need and a potential gap in clinician’s fulfilment of their moral obligations to patients. In this section, we point to some potential ways of addressing some of the limitations of informed financial consent to support more effective financial communication.

### A. *Making information more meaningful and accessible*

Improving financial communication requires considering structural forces impacting information and costs communication. In order for patients to inform themselves, they need access to good information that is understandable and relevant to the individual’s circumstances. Similarly, health professionals need access to good information to facilitate financial communication.

The business practices and models of provider organisations (and insurers) can influence financial communication by controlling what information is available to clinicians and to patients and how it is structured. Business practices may also influence financial communication through the allocation of resources, such as the availability of professionals to provide counselling and answer patients’ questions. Providers and billers may have a responsibility to provide information on the bundle of services that patients commonly require over a course of treatment, rather than per procedure billing, even if it is less convenient for an organisation to do so. It may be that we expect more transparent and organised financial information in large, corporate, vertically integrated medical conglomerates, where services are more likely to be internally bundled.

In jurisdictions with public funding and private co-payment, billing practices may be influenced by the structure of public subsidies. However, there is likely more that providers (both doctors and provider organisations) can do to improve patient cost experiences and communication. For example, negotiating with suppliers and other specialists to minimise uncertainty about costs for other elements of treatment, such as anaesthetist services. Another example is offering access to trained administrators or counsellors as standard, and ensuring those conversations can bridge financial information (and perhaps other relevant non-medical information) and the clinical options and pathways available to the patient. This may require coordination or briefing by the physician with the administrator or counsellor.

Aside from placing ethical and regulatory demands on the profession, consumer protection frameworks may also operate to address the financial information needs of patients. Because price is generally an essential term of contract, providers will generally be required to disclose to consumers a price to be paid for services. In jurisdictions such as the UK and Australia, consumer law governs contracts where a consumer pays for goods or services, even where the consumer is also a patient and the service is medical. The timing, manner, and quality of costs information and disclosure are likely to fall within consumer protections against unfair contracting and misleading or deceptive conduct. Not only is price a key term of a contract, but consumer protection may require providing information beyond merely a written contract or advertising, for example, as ‘pre-contract information’.^80^ Treatment providers (including clinicians and non-clinicians, such as clinics) must also ensure they structure costs information in a manner that is not misleading or deceptive. For example, by including information about the main characteristics of treatment, the total costs of treatment and how the price of the treatment is calculated.

### B. *Educating and guiding patients/consumers*

In recognition of the challenges of financial communication, some organisations have developed tools to help facilitate informed discussion about the financial elements of care with regard to patient needs and values (eg, Heathdirect Australia’s ‘Questions to ask your doctor’ or American Hospital Association’s ‘Understanding Healthcare Prices: A Consumer Guide’).^81^ There is also growing interest in online tools for calculating the costs of care, such as the Australian Government’s ‘Medical Costs Finder’.^82^ One of the advantages of these tools is that they do not place all the burden of disclosure on time, resource and information-constrained health professionals and can provide resources for patient to inform themselves to some extent. Another advantage is that they can educate patients and health professionals about how costs can be part of conversations about treatment. For example, a conversation guide covering how to raise financial concerns may assist patients who fear being denied or given lesser quality treatment if they ask about costs and financial information.^83^ However, information tools may not work for all individuals and evidence of low uptake by patients^84^ suggests transparency tools on their own are not a panacea for enabling informed decision making.

### C. *Using principles from informed consent*

Even without formally applying a notion of ‘informed financial consent’ (with all of its ethical and legal ramifications as discussed above), there are strong reasons for applying some of its core tenets, such as understanding and ongoing dialogue between doctor and patient, including about alternative clinical options, eliciting values, and creating space to ask questions. It is also possible to derive ideas from emerging models of informed consent that could improve decision making and the patient experience. For example, drawing on the idea from research ethics of ‘meta-consent’,^85^ a health professional could ask all patients whether or not they would like to discuss the financial implications of recommended and alternative treatments. If the answer is ‘yes’, then health professionals could ask patients a range of other open-ended questions—for example, if they have specific concerns about costs they would like to discuss; if they would like information about alternatives that differ only financially from what is being suggested; and if they would like information about potential sources of funding. If patients have questions that are highly specific, it would be quite reasonable for the health professional to refer to or suggest discussion with others, such as trained administrators, financial planners, or counsellors. Importantly, these delegates and agents must be available and accessible to patients.

There are ongoing debates about meta-consent, including its impact on ethical considerations such as autonomy^86^ and whether it can be used as a way to circumscribe a professional or ethical obligation in the clinical context. It also remains an open question what the legal implications of such an approach might be. One possibility is that doctors could be seen to fail in their legal obligations if they fail to at least ask patients whether they would like to discuss financial issues, fail to raise issues that would be considered objectively material (eg, the existence of a heavily subsidised or free alternative to a procedure that would be considered expensive by most people) or provide any false or misleading information about costs (although the latter would already be subject to consumer protection laws).

In this regard, tools developed to help facilitate informed discussion about the financial elements of care, such as the patient and consumer guides or online price estimate tools mentioned above, may support an approach to costs conversations that utilises meta-consent, buttressed by healthcare providers and professionals informing patients about and directing patients to relevant tools, and being prepared to facilitate personalisation of costs information to an individual patient’s clinical circumstances as required.

### D. *Bolstering financial communication in consumer protection law*

The application of consumer protection to the medical context is attracting increasing attention. For example, recent guidance from the UK’s Competition and Markets Authority (CMA) for fertility clinics provides some useful insight into how consumer protection may apply in instances of medical treatment. Importantly, going beyond arms–length interactions such as accuracy in advertising, the guidance highlights that consumer law governs interactions between providers and patients before and during treatment, including individually costed treatment options.^87^ While the development of guidance sheds light on how consumer protection can bolster aspects of disclosure and assist in informed consent, consumer law is more limited than an informed consent standard. For example, even where consumer law may require tailoring of costs information about an individual’s treatment options (such as following diagnostics), there is not likely to be responsibility to discuss alternatives to treatment. Further, the application of consumer protection should not obscure the importance of the doctor–patient relationship and the need to address the special vulnerabilities of patients that make them a unique category of consumers.

## V. CONCLUSION

Good and timely costs information is generally necessary, but not always sufficient to enable patients to make informed decisions about their care. Complexity, uncertainty, and individual variance in healthcare costs tend to make disclosure and financial transparency challenging. Disclosure of patient-specific costs may be adequate in some, simple circumstances, but it may not fully satisfy doctors’ moral obligations to their patients in others. In particular, mere disclosure does not satisfy the criteria for genuine ‘informed consent’ or ‘shared decision-making’, in which discussions of cost need to be integrated with discussions about clinical risks and benefits, enabling patients to determine not only what they can afford, but also what represents the most value for money to them. Thus, even if financial information can be effectively disclosed, this may fail to achieve the ethical, economic and legal goals that underpin and justify disclosure requirements.

Simply putting the ‘financial’ into ‘informed consent’ and invoking an informed consent standard for cost information as a complete approach to financial communication in the medical context has several ethical, legal, and practical difficulties, including determining patient values regarding financial risks, harms, and benefits in order to tailor the consenting process; identifying what might count as a ‘cost’; determining the materiality of costs information; in obtaining costs information about alternative treatments or providers; managing assumptions about affordability; managing unwillingness of patients or clinicians to engage in discussions about costs; managing lack of expertise to assess financial capacity; and navigating the boundary between discussing the financial aspects and implications of treatment, with providing financial advice. Furthermore, informed financial consent also faces the ongoing challenges of obtaining informed consent based on information about non-financial risks and benefits.

But even without formally applying a notion of ‘informed financial consent’ (with all of its ethical and legal ramifications), it is possible to derive ideas from informed consent that could improve decision making and the patient experience. It is clear that practitioners (and their delegates) have a unique role to play in bridging the gap between disclosure and enabling informed (financial) decision making, including in contextualising costs among clinical options for individual patients and being a touchpoint to link patients with resources. The next step is to consider whether a consent-oriented approach should be considered part of, or additional to, consent to medical treatment itself. The extent to which consent to costs might be considered part of consent to treatment will likely depend on the legal bases, structures, and goals of professional regulation in a particular jurisdiction.

Whether or not our suggested approach to financial communication is integrated into consent to treatment, financial communication between doctors and patients must be part of a multi-faceted approach. Any ethical and regulatory demands on professionals regarding informed and shared decision making must be cognisant of the influence of non-clinical providers and other structural forces on discharging such obligations. There is need to incentivise providers to make good, timely and meaningful information available to individuals and practitioners, and recent developments in the UK suggest that the consumer law may be an effective tool to align provider behaviour with good informing processes. More broadly, as out-of-pocket costs associated with health care will likely continue to grow, there is need to make financial expertise and counselling accessible to patients in healthcare contexts with high potential for financial harm.

There is no way of escaping the need to discuss the financial aspects of care with patients. This cannot, however, be achieved simply by invoking legal and ethical ideas of ‘informed financial consent’ without considering the challenges that surround consent in general and the specific challenges related to applying notions of consent to financial communication.

## Funding 

This research was funded by the National Health and Medical Research Council (NHMRC Ideas Grant APP1181401).

## Footnotes

1 See R Harvey, ‘Out-of-Pocket Payments for Health Care—Finding a Way Forward’ in Australian Department of Parliamentary Services, *Parliamentary Library Briefing Book - 44th Parliament of Australia* (December 2013); V Arora, C Moriates and N Shah, ‘The Challenge of Understanding Health Care Costs and Charges’ (2015) 17 AMA Journal of Ethics 1046; R Gupta, C Tsay and RL Fogerty, ‘Promoting Cost Transparency to Reduce Financial Harm to Patients’ (2015) 17 AMA Journal of Ethics 1073; L Russell and J Doggett, *A Road Map for Tackling Out-of-Pocket Health Care Costs* (White Paper, February 2019); GL Smith and others, ‘Financial Burdens of Cancer Treatment: A Systematic Review of Risk Factors and Outcomes’ (2019) 17 Journal of the National Comprehensive Cancer Network 1184; R Crowley and others, ‘Envisioning a Better U.S. Health Care System for All: Coverage and Cost of Care’ (2020) 172 Annals of Internal Medicine S7; EJ Callander, ‘Out‐of‐pocket Fees for Health Care in Australia: Implications for Equity’ (2023) Medical Journal of Australia <https://www.mja.com.au/journal/2023/218/7/out-pocket-fees-health-care-australia-implications-equity> accessed 5 July 2023.

2 See Executive Order 13877 [2019] Improving price and quality transparency in American healthcare to put patients first, 84 FR 30849; Australian Government Department of Health and Aged Care, ‘Informed Financial Consent Guide Launched’ (*Australian Government Department of Health and Aged Care*, 23 July 2019) <https://www.health.gov.au/ministers/the-hon-greg-hunt-mp/media/informed-financial-consent-guide-launched> accessed 13 June 2023; MC Politi and others, ‘Discussing Cost and Value in Patient Decision Aids and Shared Decision Making: A Call to Action’ (2023) 8 MDM Policy & Practice 1.

3 For example, an Executive Order by the President of the United States in 2019 called for improving price transparency and costs information before making medical decisions. See n 2.

4 NN Sawicki, ‘Modernizing Informed Consent: Expanding the Boundaries of Materiality’ (2016) 2016 University of Illinois Law Review 821.

5 Medical Board of Australia, *Good Medical Practice: A Code of Conduct for Doctors in Australia* (October 2020), s 4.5.3.

6 ibid.

7 General Medical Council, *Guidance on Professional Standards and Ethics for Doctors Decision Making and Consent* (2020).

8 Medical Council of New Zealand, ‘Your Rights as a Patient’ (2019) <https://www.mcnz.org.nz/support/support-for-patients/your-rights-as-a-patient/> accessed 10 July 2023.

9 See eg, D Currow and S Aranda, ‘Financial Toxicity in Clinical Care Today: A “Menu without Prices”’ (2016) 204 Medical Journal of Australia 397; KA Schulman and BD Richman, ‘Informed Consent as a Means of Acknowledging and Avoiding Financial Toxicity as Iatrogenic Harm’ (2022) 24 AMA Journal of Ethics 1063.

10 For eg, the Australian Medical Association recommend that financial consent be a part of clinical care of patients: Australian Medical Association, *Informed Financial Consent* (Position Statement, June 2015) <https://www.ama.com.au/articles/informed-financial-consent-2015> accessed 1 December 2022.

11 See eg, Australian Government Department of Health and Aged Care (n 2).

12 Australian Government Senate Community Affairs Legislation Committee, *Report—Health Legislation Amendment (Gap Cover Schemes) Bill 2000* (Commonwealth of Australia 2000) <https://www.aph.gov.au/parliamentary_business/committees/senate/community_affairs/completed_inquiries/1999-02/gapcover/report/index> 8 August 2023.

13 Australian Commission on Safety and Quality in Health Care, *AS18/10: Informed financial consent* (November 2021).

14 Australian Government Department of Health and Aged Care (n 2).

15 For eg, the Australian Medical Association recommend that financial consent be a part of clinical care of patients: Australian Medical Association, *Informed Financial Consent* (Position Statement, June 2015) <https://www.ama.com.au/articles/informed-financial-consent-2015> accessed 1 December 2022.

16 Health Care Complaints Commission, Professional Standards Committee Inquiry (NSW), *Complaint against Dr Francis Cheuk Kin Chu*, (Ref: H18/10352 DD20/09534, 2020). Note that it was relevant for the Committee that the doctor knew that the patient had limited financial capacity (elderly and uninsured).

17
*Adelaide Anaesthetic Services (Reg) v John Smith* No 93/17705  [1994] SASC 5019; [1994] SAMC 1, as referred to in J Germov, ‘Medi-Fraud, Managerialism and the Decline of Medical Autonomy: Deprofessionalisation and Proletarianisation Reconsidered’ (1995) 31 The Australian and New Zealand Journal of Sociology 51.

18
*Adelaide Anaesthetic Services (Reg) v John Smith* No 93/17705  [1994] SASC 5019; [1994] SAMC 1. Notably, this case does not mention ‘informed financial consent’ or ‘informed consent’, nor does it especially deal with the doctor-patient relationship between the parties. Rather, the common law implies the otherwise unagreed-upon price term in the contract for supply of anaesthetic services in conjunction with surgery.

19
*Adelaide Anaesthetic Services (Reg) v John Smith* No 93/17705  [1994] SASC 5019; [1994] SAMC 1.

20 J Germov, ‘Medi-Fraud, Managerialism and the Decline of Medical Autonomy: Deprofessionalisation and Proletarianisation Reconsidered’ (1995) 31 The Australian and New Zealand Journal of Sociology 51.

21 See eg, BD Richman, MA Hall and KA Schulman, ‘Overbilling and Informed Financial Consent–A Contractual Solution’ (2012) 367 New England Journal of Medicine 396; D Currow and S Aranda, ‘Financial Toxicity in Clinical Care Today: A “Menu without Prices”’(2016) 204 Medical Journal of Australia 397; C Thamm and others, ‘Exploring the Role of General Practitioners in Addressing Financial Toxicity in Cancer Patients’ (2022) 30 Supportive Care in Cancer 457; Schulman and Richman (n 9).

22 See Richman, Hall and Schulman ibid.

23 BD Richman, MA Hall and KA Schulman, ‘The No Surprises Act and Informed Financial Consent’ (2021) 385 The New England Journal of Medicine 1348.

24 See eg, Australian Government Productivity Commission, *Performance* of *Public and Private Hospitals* (Commonwealth of Australia 2009) <https://www.pc.gov.au/inquiries/completed/hospitals/report/hospitals-report.pdf> accessed 8 August 2023.

25 New South Wales Government, *Policy Directive—Charging Arrangements for Hospitals and Other Health Services. Financial Accounting, Policy, Insurance and Revenue* (July 2022) <https://www1.health.nsw.gov.au/pds/ActivePDSDocuments/PD2022_024.pdf> accessed 8 August 2022.

26 Australian Medical Association, *Informed Financial Consent—a collaboration between doctors and patients* (AMA 2020) 11.

27 DE Hall, AV Prochazka and AS Fink, ‘Informed Consent for Clinical Treatment’ (2012) 184 CMAJ: Canadian Medical Association Journal 533.

28 See RD Nipp, EM Sonet and GP Guy, ‘Communicating the Financial Burden of Treatment with Patients’ (2018) American Society of Clinical Oncology Educational Book 524; A Agarwal and others, ‘Discussion of Costs and Financial Burden in Clinical Practice: A Survey of Medical Oncologists in Australia’ (2022) 17 PLoS One e0273620.

29 For the purposes of this article, we remain agnostic as to whether current ethical and legal understandings of ‘consent to treatment’ should be explicitly expanded to include information about costs or whether consent to medical treatment and consent to cost are separate obligations.

30 See MA Hall and others, *Solving Surprise Medical Bills*, The Schaeffer Initiative for Innovation in Health Policy, A Brookings Institution–USC Schaeffer Center Partnership (White Paper, October 2016); Richman, Hall and Schulman (n 23); Richman, Hall and Schulman (n 21).

31 See YN Wong and others, ‘Understanding How Out-of-Pocket Expenses, Treatment Value, and Patient Characteristics Influence Treatment Choices’ (2010) 15 The Oncologist 566; C Exley and others, ‘Beyond Price: Individuals’ Accounts of Deciding to Pay for Private Healthcare Treatment in the UK’ (2012) 12 BMC Health Services Research 53; SY Zafar and others, ‘The Utility of Cost Discussions between Patients with Cancer and Oncologists’ (2015) 21 The American Journal of Managed Care 607; Australian Government Department of Health and Aged Care (n 2); Executive Order 13877 [2019] Improving price and quality transparency in American healthcare to put patients first, 84 FR 30849; HS Smith, ‘How Should Economic Value Be Considered in Treatment Decisions for Individual Patients?’ (2021) 23 AMA Journal of Ethics 607.

32 AL Meluch and WH Oglesby, ‘Physician–Patient Communication Regarding Patients’ Healthcare Costs in the USA: A Systematic Review of the Literature’ (2015) 8 Journal of Communication in Healthcare 151.

33 ‘Financial toxicity’ describes the impact of out-of-pocket costs associated with treatment that can diminish quality of life and inhibit delivery of quality care. The term has been used to include both objective financial burden and subjective experiences of financial distress in cancer care and more broadly: SY Zafar and AP Abernethy, ‘Financial Toxicity, Part I: A New Name for a Growing Problem’ (2013) 27 Oncology (Williston Park, NY) 80.

34 See Cancer Council, *Cancer Charities Call for Better Disclosure on Cost of Treatment* (Cancer Council, 15 November 2018) <https://www.cancer.org.au/media-releases/2018/cancer-charities-call-for-better-disclosure-on-cost-of-treatment> accessed 29 June 2023; K Chalmers, AG Elshaug and S Larkin, ‘First Steps towards Price Transparency: Comparability of Online Out-of-Pocket Tools from Australian Private Health Funds’ (2020) 44 Australian Health Review 347; Schulman and Richman (n 9); Richman, Hall and Schulman (n 23).

35 S Scheetz and MH Chin, ‘Ethical Dimensions of Pricing Transparency’ (2022) 24 AMA Journal of Ethics 1031.

36 See eg, C Whaley and A Frakt, ‘If Patients Don’t Use Available Health Service Pricing Information, Is Transparency Still Important?’ (2022) 24 AMA Journal of Ethics 1056; Australian Commission on Safety and Quality in Health Care, *Understanding My Healthcare Rights: A Guide for Consumers* (ACSQHC 2020).

37 K Campbell and K Parsi, ‘A New Age of Patient Transparency: An Organizational Framework for Informed Consent’ (2017) 45 Journal of Law, Medicine & Ethics 60.

38 OAJ Mascarenhas, R Kesavan and MD Bernacchi, ‘On Reducing Information Asymmetry in U.S. Health Care’ (2013) 30 Health Marketing Quarterly 379.

39 JC Newton and others, ‘“…If I Don’t Have That Sort of Money Again, What Happens?”: Adapting a Qualitative Model to Conceptualise the Consequences of out-of-Pocket Expenses for Cancer Patients in Mixed Health Systems’ (2020) 44 Australian Health Review 355.

40 Campbell and Parsi (n 37); N Shah, ‘Physicians’ Role in Protecting Patients’ Financial Well-Being’ (2013) 15 AMA Journal of Ethics 162.

41 Richman, Hall and Schulman (n 21).

42 Focus on price transparency has been described as a ‘national movement’ in the USA, fueled by the increasing availability of online tools for patients: Arora, Moriates and Shah (n 1) 1048.

43 In *Adelaide Anaesthetic Services (Reg) v John Smith* No. 93/17705  [1994] SASC 5019; [1994] SAMC 1, an anaesthetist failed to inform the patient of out-of-pocket expenses. The Magistrate implied the reasonable fee as the amount offered for the service by the Australian government subsidy (Medicare Benefits Schedule). Thus, the court’s implied price term in the contract between the provider and patient meant that the patient did not owe the anaesthetist the additional private fee.

44 GK Hadfield, R Howse and MJ Trebilcock, ‘Information-Based Principles for Rethinking Consumer Protection Policy’ (1998) 21 Journal of Consumer Policy 131.

45 Whaley and Frakt (n 36).

46 C Stewart and I Kerridge, ‘The Three Functions of Consent in Neurosurgery’ in S Honeybul (ed), *Ethics in Neurosurgical Practice* (CUP 2020), 29-38.

47 ibid.

48 For healthcare professionals, consent may be said to perform an instrumental function in the avoidance of legal liability.

49 Stewart and Kerridge (n 46).

50 ibid.

51 See also Hall, Prochazka and Fink (n 27); SN Whitney, AL McGuire and LB McCullough, ‘A Typology of Shared Decision Making, Informed Consent, and Simple Consent’ (2004) 140 Annals of Internal Medicine 54.

52 See TC Hoffmann and others, ‘Shared Decision Making: What Do Clinicians Need to Know and Why Should They Bother?’ (2014) 201 Medical Journal of Australia 35. <https://www.mja.com.au/journal/2014/201/1/shared-decision-making-what-do-clinicians-need-know-and-why-should-they-bother> accessed 17 May 2023.

53 See E Jackson, ‘Challenging the Comparison in Montgomery Between Patients and “Consumers Exercising Choices”’(2021) 29 Medical Law Review 595.

54 See eg, Australian Medical Association (n 10). The Australian Medical Association supports doctor-patient costs conversations in principle but does not support a legally enshrined obligation with sanctions for non-compliance.

55 Whaley and Frakt (n 36).

56 Schulman and Richman (n 9).

57 ibid.

58 Though in some healthcare contexts, such as the US system of networked care, ‘shoppability’ also occurs at the point of obtaining insurance.

59 United Kingdom Competition and Markets Authority, *Guidance for Fertility Clinics on Consumer Law: Helping Fertility Clinics Comply with Their Consumer Law Obligations* (CMA127, June 2021) para 1.6.

60 C Mayes, J Williams and W Lipworth, ‘Conflicted Hope: Social Egg Freezing and Clinical Conflicts of Interest’ (2018) 27 Health Sociology Review 45.

61 Hall, Prochazka and Fink (n 27).

62 B Davies and J Parker, ‘Doctors as Appointed Fiduciaries: A Supplemental Model for Medical Decision-Making’ (2022) 31 Cambridge Quarterly of Healthcare Ethics 23.

63 CR Vernazza and others, ‘Introducing High-Cost Health Care to Patients: Dentists’ Accounts of Offering Dental Implant Treatment’ (2015) 43 Community Dentistry and Oral Epidemiology 75.

64 ibid. See also PM Rosoff, ‘Who Should Ration?’ (2017) 19 AMA Journal of Ethics 164.

65 See S Weiner, ‘“I Can’t Afford That!”’ (2001) 16 Journal of General Internal Medicine 412.

66 Including comparative waiting times: Currow and Aranda (n 9). Further, a complaint prosecuted in an Australian Medical Professional Standards Committee in 2020 found a doctor guilty of unsatisfactory professional conduct for failing to provide adequate information to a cancer patient about the possibility of surgery being performed at the public hospital: Health Care Complaints Commission, Professional Standards Committee Inquiry (NSW), *Complaint against Dr Francis Cheuk Kin Chu*, (Ref: H18/10352 DD20/09534, 2020).

67 Whether physicians have a duty to disclose more cost-effective ways to access medicines has been considered elsewhere: N Ghinea, ‘Physicians’ Legal Duty to Disclose More Cost-Effective Treatment Options: An Examination of Australian Civil Law Applied to Personal Importation’ (2023) 47 Australian Health Review 314.

68 Medical Board of Australia, *Guidelines for Registered Medical Practitioners Who Perform Cosmetic Medical and Surgical Procedures* (Medical Board of Australia 2019).

69 ibid.

70 See Currow and Aranda (n 9); Cancer Council of Australia, Standard for Informed Financial Consent (April 2020) <https://www.cancer.org.au/assets/pdf/standard_for_informed_financial_consent> accessed 15 June 2023.

71 N Bhatia and L Porceddu, ‘Emptying the Nest Egg to Fill the Nursery: Early Release of Superannuation to Fund Assisted Reproductive Technology’ 2021 University of New South Wales Law Journal 513.

72 An Australian Financial Services Licence issued by the Australian Securities and Investments Commission (ASIC) may be required under ch 7 (s 911A) of the *Corporations Act**2001* (Cth).

73 Medical Board of Australia (n 68).

74 M Pisu and others, ‘Perspectives on Conversations About Costs of Cancer Care of Breast Cancer Survivors and Cancer Center Staff’ (2019) 170 Annals of Internal Medicine S54; SY Zafar and others, ‘Cost-Related Health Literacy: A Key Component of High-Quality Cancer Care’ (2015) 11 Journal of Oncology Practice 171.

75 Zafar and others (n 31).

76 See Sawicki (n 4); J O’Neill, ‘Materiality of Conflict of Interest in Informed Consent to Medical Treatment in the United Kingdom’ (2022) 32 Ethics & Behavior 375, which argues that disclosure of financial interest should be included in the duty to warn (UK).

77
*Rogers v Whitaker* (1992) 175 CLR 479. In Australia, a doctor’s duty to provide information to patients before undertaking a medical procedure derives principally from the law of negligence, but the law of trespass and contract are also relevant and the common law principles have been stated in legislation in some jurisdictions.

78 See eg, *Montgomery v Lanarkshire Health Board* [2015] UKSC 11; *Rogers v Whitaker* (1992) 175 CLR 479.

79 PA Ubel, AP Abernethy and SY Zafar, ‘Full Disclosure—Out-of-Pocket Costs as Side Effects’ (2013) 369 The New England Journal of Medicine 1484.

80 For eg, as in the United Kingdom Competition and Markets Authority (n 39). Though as E Jackson points out, pre-contractual information disclosures to consumers are different to the process of gaining information consent for treatment, including in terms of quality of information and the dynamics between the parties: Jackson (n 53).

81 Health Direct Australia, ‘Questions to ask your doctor’ (February 2023) <https://www.healthdirect.gov.au/questions-to-ask-your-doctor> accessed 9 August 2023; Healthcare Financial Management Association, ‘Understanding Healthcare Prices: A Consumer Guide’ (2018) <https://www.aha.org/system/files/2018-04/14transparency-consumerguide.pdf> accessed 10 July 2023.

82 Australian Government Department of Health, ‘Medical Costs Finder’ (11 November 2022) <https://www.health.gov.au/resources/apps-and-tools/medical-costs-finder> accessed 8 August 2023.

83 See Zafar and others (n 31).

84 A Zhang and others, ‘The Impact of Price Transparency on Consumers and Providers: A Scoping Review’ (2020) 124 Health Policy 819; Whaley and Frakt (n 36).

85 T Ploug and S Holm, ‘Meta Consent: A Flexible and Autonomous Way of Obtaining Informed Consent for Secondary Research’ (2015) 350 British Medical Journal (Clinical research ed.) h2146.

86 See NC Manson, ‘The Biobank Consent Debate: Why “Meta-consent” is Not the Solution?’ (2019) 45 Journal of Medical Ethics 291; T Ploug and S Holm. ‘The Biobank Consent Debate: Why ‘Meta-Consent’ is Still the Solution!’ (2019) 45 Journal of Medical Ethics 295.

87 United Kingdom Competition and Markets Authority (n 39); United Kingdom Competition and Markets Authority, *Fertility Treatment: A Guide to Your Consumer Right*s (CMA142, June 2021).

